# Differential placental DNA methylation of *VEGFA* and *LEP* in small-for-gestational age fetuses with an abnormal cerebroplacental ratio

**DOI:** 10.1371/journal.pone.0221972

**Published:** 2019-08-30

**Authors:** Iris Bekkering, Mariëtte Leeuwerke, Jozien C. Tanis, Mirthe H. Schoots, Rikst Nynke Verkaik-Schakel, Torsten Plösch, Caterina M. Bilardo, Jasper J. H. Eijsink, Arend F. Bos, Sicco A. Scherjon

**Affiliations:** 1 Department of Obstetrics and Gynecology, University Medical Center Groningen, University of Groningen, Groningen, The Netherlands; 2 Department of Neonatology, Beatrix Children’s Hospital, University Medical Center Groningen, University of Groningen, Groningen, The Netherlands; 3 Department of Pathology, University Medical Center Groningen, University of Groningen, Groningen, The Netherlands; Texas A&M University College Station, UNITED STATES

## Abstract

**Background:**

In Fetal Growth Restriction ‘fetal programming’ may take place via DNA methylation, which has implications for short-term and long-term health outcomes. Small-for-gestational age fetuses are considered fetal growth restricted, characterized by brain-sparing when fetal Doppler hemodynamics are abnormal, expressed as a cerebroplacental ratio (CPR) <1. We aimed to determine whether brain-sparing is associated with altered DNA methylation of selected genes.

**Methods:**

We compared DNA methylation of six genes in 41 small-for-gestational age placentas with a normal or abnormal CPR. We selected *EPO*, *HIF1A*, *VEGFA*, *LEP*, *PHLDA2*, and *DHCR24* for their role in angiogenesis, immunomodulation, and placental and fetal growth. DNA methylation was analyzed by pyrosequencing.

**Results:**

Growth restricted fetuses with an abnormal CPR showed hypermethylation of the *VEGFA* gene at one CpG (*VEGFA*-309, p = .001) and an overall hypomethylation of the *LEP* gene, being significant at two CpGs (*LEP*-123, p = .049; *LEP*-51, p = .020). No differences in methylation were observed for the other genes.

**Conclusions:**

*VEGFA* and *LEP* genes are differentially methylated in placentas of small-for-gestational age fetuses with brain-sparing. Hypermethylation of *VEGFA*-309 in abnormal CPR-placentas could indicate successful compensatory mechanisms. Methylation of *LEP*-51 is known to suppress *LEP* expression. Hypomethylation in small-for-gestational age placentas with abnormal CPR may result in hyperleptinemia and predispose to leptin-resistance later in life.

## Introduction

Fetal Growth Restriction (FGR) complicates 5 to 10% of all pregnancies and is a major risk factor for fetal and neonatal morbidity and mortality. Fetuses with an estimated fetal weight <10^th^ percentile for gestational age are commonly classified as small-for-gestational age (SGA), however, many of these fetuses do reach their biological potential and are constitutionally small. Doppler assessment of fetal hemodynamics discerns between SGA and FGR. The fetal vascular response to placental insufficiency, resulting in FGR is called “brain-sparing”, a circulatory redistribution in which blood flow is preferentially shunted to the brain [1]. Brain-sparing can be quantified with the cerebroplacental ratio (CPR), calculated as Middle Cerebral Artery (MCA) Pulsatility Index (PI) divided by Umbilical Artery (UmbA) PI. As the name suggests, brain-sparing is thought to be a beneficial adaptive mechanism to prevent brain damage [2,3]. However, recent studies suggest that FGR fetuses with brain-sparing might be at an increased risk for neurodevelopmental and behavioral deficits, such as reduced gross and fine motor skills, and a reduced intelligence quotient [4].

Brain-sparing is indicating an adverse intrauterine condition that can lead to ‘fetal programming’, which has implications for short-term as well as long-term health. This programming can occur via alterations in DNA methylation, an epigenetic mechanism, which regulates gene expression. Differential methylation and/or gene expression has been reported in FGR for various genes, including those encoding for erythropoietin (*EPO*), hypoxia inducible factor 1 alpha subunit (*HIF1A*), vascular endothelial growth factor A (*VEGFA*), leptin (*LEP*), pleckstrin homology-like domain family A member 2 (*PHLDA2*) and 3-β-hydroxysterol delta-24-reductase (*DHCR24*) [5–8]. In this study, we focused on methylation of these genes in the placenta as they have profound roles in angiogenesis, neurodevelopment, immunomodulation and placental and fetal growth.

The methylation status of angiogenic genes in the placenta is of interest because of the possible hypoxic state that the FGR fetus can encounter. This hypoxic stress drives activation of angiogenic compensatory mechanisms such as *EPO*, *HIF1A* and *VEGFA*. *EPO* is expressed in the placenta and supports the fetal condition by e.g. regulating fetal red blood cell production, promoting erythroid differentiation and initiating hemoglobin syntheses [9]. *HIF1A* is a pro-angiogenic regulator in the anti-hypoxic response [10]. In response to hypoxia, *HIF1A* binds to the enhancer region of *EPO* and drives its expression [11]. The effect of *HIF1A* is favorable in the acute phase but can become pathological if prolonged, e.g. in placenta dysfunction in preterm preeclampsia (PE) [12]. *VEGFA* is an important regulator of angiogenesis which stimulates placental endothelial cell proliferation [8]. Increased *VEGFA* expression has been found in FGR placentas [8]. In preterm PE placentas, hypomethylation of *VEGFA* has been reported, resulting in upregulated expression [13]. We hypothesized that methylation of *EPO*, *HIF1A* and *VEGFA* in the placenta may play a role in the response to hypoxia and differs for fetuses that show brain-sparing.

With respect to the presence of brain-sparing, we expect to see methylation changes in *LEP*, *PHLDA2* and *DHCR24*, as these genes are associated with neurodevelopment. *LEP*, commonly known as the “hormone of energy expenditure”, is an important metabolic hormone which is highly expressed in the placenta and regulates placental and fetal growth [14]. In the fetus, *LEP* is suggested to play a role in fetal growth, angiogenesis, hematopoiesis, brain development, and immunity [14]. Recent studies have associated changes in *LEP* methylation and expression with fetal growth, SGA, FGR, and neurodevelopmental outcome [6,7,15–19]. So far, methylation of *LEP* and its relation to the CPR has not been investigated. *LEP* is a possibly important hormone to investigate in FGR since short- and long-term adverse effects of FGR include obesity, metabolic syndrome, and diabetes. Altered methylation and/or expression of *LEP* may contribute to the susceptibility of FGR infants to these diseases. *PHLDA2* is a well-known maternally expressed imprinted gene, which represses placental growth. This highlights the important balance of *PHLDA2* needed for optimal fetal growth. Various studies have reported overexpression of this gene in SGA placentas and association with (low) birth weight, while others did not report a significant difference [20,21]. Overexpression has also been associated with reduced fetal movements followed by SGA, further emphasizing the clinical relevance of *PHLDA2* [22].

*DHCR24* encodes for an enzyme that catalyzes the final step in the synthesis of cholesterol. Cholesterol is essential for cell membranes and critical for fetal development, particularly brain development [23]. Furthermore, *DHCR24* has an anti-apoptotic effect on neurons, is thought to be neuroprotective, and its expression is down-regulated in SGA placentas [24].

In this study, our aim was to investigate whether methylation of selected angiogenic and neurodevelopmental genes differs between SGA placentas with normal or abnormal Doppler fetal hemodynamics, expressed as a normal/abnormal CPR. This seems to be the case for *VEGFA* and *LEP* methylation. It is of clinical importance to discern the presence of brain-sparing with the CPR in these SGA placentas as this indicates a compensatory mechanisms to placental insufficiency which is considered to give rise to fetal programming.

## Materials and methods

### Study population

We performed a prospective observational cohort study from June 2012 until September 2014 at the Obstetrics and Neonatology Departments of the University Medical Center Groningen (UMCG). The study was approved by the Institutional Review Board of the UMCG and written informed consent was obtained from the mothers of the 48 SGA fetuses that were included [25]. SGA was defined as an estimated fetal weight below the 10^th^ percentile or deflecting fetal growth of at least 30 percentiles. Exclusion criteria adopted were: multiple pregnancy; structural and chromosomal abnormalities; and intrauterine infection. All deliveries and admissions to the neonatal ward/neonatal intensive care unit took place in the UMCG. In our study 41 of the SGA cases were included, based on availability of both placental tissue and CPR data. Embedded paraffin slices of placental tissue from this cohort were stored at the UMCG. Usage of the samples was approved by the UMCG Medical Ethical Committee in line with the code of conduct for responsible use.

### Selection of genes and primer design

We selected biologically relevant genes involved in placental/fetal growth, angiogenesis or neurodevelopment. The genomic target region (Table 1) was chosen based on existing literature regarding regulation by methylation and/or association with SGA, FGR, or PE.

**Table 1:** PCR primer sequences accompanied by sequencing primers and their genomic location (*Homo sapiens* GRCh38.p7 primary assembly).

Gene	Primers	Sequence to Analyze	Genomic Region
***EPO***	PCR Forward: 5'- GGGGGTAGGGGTTGTTATTTGTATG -3' PCR Reverse: 5'-Biotin-CCCAAACCTCCTACCCCTACTCTAACC-3' Sequencing Primer 5'- GGGTTGTTATTTGTATGTG -3'	TGYGTGYGYGGGTGGGGGTGGGGGAGAGGTTGTGTGYGTGAGGGGTYGTTAGGGGTAGGGGTTATTYGGGGTTAGAGTAGGGGTAGGA	Chromosome 7: 100720774–100720822
***HIF1A***	PCR Forward: 5'- AGGAGGTTAGTTGAGGTATAGTTGG-3' PCR Reverse: 5'- Biotin -CACCCCCATCTCCTTTCT-3' Sequencing Primer 5'- GTTGAGGTATAGTTGGGA -3'	YGGGTTGYGAYGTTTAYGTGTTYGTTTGTGTTTAGYGGYGGAGGAAAGAGAAAGGAGATGGGGG	Chromosome 14: 61695200–61695263
***VEGFA***	PCR Forward: 5'-GGGAGTAGGAAAGTGAGGT-3' PCR Reverse: 5'-Biotin-TTCCCCTACCCCCTTCAATAT -3' Sequencing Primer 5'-AGTAGGAAAGTGAGGTTA-3'	YGTGYGGATAGGGTTTGAGAGTYGTTTTTTTTTTGTTAGGAATATTGA	Chromosome 6: 43769854–43769901
***LEP***	PCR Forward: 5'- GGTGTATATTGAGGGTTTAGGGTTAGTA -3' PCR Reverse: 5'- Biotin-CCATACCTACCCCCCCCTCTTATAAC -3' Sequencing Primer 1 5'- GGTTTAGGGTTAGTAGT -3' Sequencing Primer 2 5'- GGGAGTTGGAGTTAGAAATG -3'	Sequence 1 YGTTYGGTAYGTYGTTATTTTGAGGGGYGGGGYGGGAGTTGGYGTTAGAAATG Sequence 2: YGTYGGGGTTTGYGGGGTAGTTGYGTAAGTTGTGATYGGGTYGTTATAAGAGGGGYGGGTAGGTATGGAGTTT	Chromosome 7: 128241151–128241276
***PHLDA2***	PCR Forward: 5'-GTTTTTGGGAGGTTTTGGGAAAGGT-3' PCR Reverse: 5'-Biotin-AATCCAAACCCCCTTCCTTACTCC-3' Sequencing Primer 5'-GGTTTTGGGAAAGGTT-3'	TTYGAATAGGAYGGAAYGGTTAGGGTTYGGGAGTAAGGAA GGGGGTTT	Chromosome 11: 2929921–2929955
***DHCR24***	PCR Forward: 5'-Biotin-GGGGTGGGTAGTTGGGATTTA-3' PCR Reverse: 5'-TACAAAAATTACCCCCAAATTTCCATCCTC-3' Sequencing Primer 5'-CCAAATTTCCATCCTCC -3'	RCRCCCRAAAACCRCCTATCCRACCAAAAAATAACTAAC	Chromosome 1: 54887442–54887619

The analyzed *EPO* promoter region has been reported to repress transcription via hypermethylation in cancer [26] and contains a hypoxia-inducible factor-1B binding site [27]. The selected *HIF1A* promoter region contains a hypoxia response element that is thought to facilitate positive *HIF1A* autoregulation via hypomethylation [28], and a kaiso binding site-3 suggested to mediate methylation-dependent *HIF1A* repression [27]. The *VEGFA* region has been studied by Sundrani et al. who reported hypomethylation in PE placentas [13]. Our *LEP* region overlaps the promoter region investigated by Lesseur and colleagues which is regulated by methylation [29–31]. Hypermethylation of the selected *PHLDA2* promoter region silences expression [32]. For *DHCR24* we selected a cytosine-phosphate-guanosine (CpG) dinucleotides-rich promoter region reported to regulate transcription via methylation [33].

All PCR and sequencing primers (Table 1) were designed with the PyroMark Assay Design software (Qiagen, Hilden, Germany) and a gene specific biotinylated reversed/forward primer approach was used to label the PCR product with biotin. For bisulfite amplification we prepared a mastermix containing 12.5 μL HotStarTaq DNA Polymerase, 10.5 μL sterile water and a 1 μL mix of forward and reverse primer (10:10 μM) per 1 μL bisulfite template. We included a mastermix negative control to check for contamination. Cycling conditions on the T100 Thermal Cycler (Bio-Rad, Hercules, CA) were the same for all assays except for *HIF1A*: 95°C for 15 min, 45 cycles of 94°C for 30 s, 58°C (*HIF1A* 56°C) for 30 s, 72°C for 30 s, followed by a final step of 72°C for 7 min and stored at 4°C. A DNA ladder and 3–5μL of each PCR product was loaded and run on 2% agarose gel with ethidium bromide staining to visualize presence or absence of PCR products and contamination.

Placenta was processed by investigators unaware of the clinical data. First, the fetal side was selected by our pathologist on a hematoxylin-eosin stained section. Cores with a diameter of 1mm from the fetal side were used for DNA isolation with the QIAamp DNA FFPE (Formalin-Fixed, Paraffin-Embedded) Tissue Kit (Qiagen). The FFPE tissue protocol is designed for sections of 5–10μm and was adapted for 1mm cores. This entailed vortexing the samples at step 3 for 1 minute and crushing the cores manually with a pipet tip. Quality and concentration of the isolated DNA was checked with the NanoDrop®ND-1000 spectrophotometer (Thermo Fisher Scientific, Waltham, MA). Samples were stored at -20°C.

For bisulfite conversion, we used the EZ methylation Gold-Kit (Zymo Research, Irvine, CA) according to the supplier’s protocol with an additional 30s centrifuge round at full speed after step 8. Samples were divided in two separate batches for adequate desulphonation time, random and blinded. In total, 500 nanograms of each DNA sample was bisulfite converted for subsequent analysis by pyrosequencing.

### Quantification of methylation via pyrosequencing

We used the PyroMarkQ48 Autoprep (Qiagen) for pyrosequencing, according to the supplier’s protocol. Methylation levels were analyzed with PyroMark Q48 Advanced Software (Qiagen). Each of the CpG sites were quality control checked and the percentage of DNA methylation at a given CpG calculated. CpGs that passed the quality check were included in data-analysis. Excluded samples were repeated.

### Doppler ultrasound

Doppler parameters of the UmbA and MCA were measured once a week prior to maternal admission to the hospital and twice a week for the duration of admission by two experienced operators, as described elsewhere [25]. The CPR was calculated as MCA-PI divided by UmbA-PI. CPR data was transformed to z-scores as appropriate for the gestational age-specific mean [25]. A CPR of <1 was considered abnormal. For data analysis, we used the last measured CPR before delivery, with a maximum of seven days prior to birth.

### Statistical analysis

We assessed normality with the Shapiro-Wilk test, data with a Shapiro-Wilk p-value of < .05 was considered to follow a non-normal distribution. A parametric or non-parametric approach was chosen as appropriate for the data. Continuous variables were compared using Student’s T-test or Mann-Whitney U test, categorical data with Chi-square or Fisher’s Exact test, and correlations with Pearson’s Test or Spearman’s Rho. We used simple and multiple linear regression analyses to analyze independent associations between variables. For this, non-normal distributed data were logarithmically transformed. A p-value of < .05 (two-tailed) was considered statistically significant. Descriptive statistics are presented as mean (SD), median (range), or frequency (%). We used the software Statistical Package for the Social Sciences (SPSS) 23.0 for statistical analyses.

## Results

### Characteristics of the study population

The study cohort consisted of 48 SGA patients of whom we selected 41 based on availability of both placenta material and CPR data. Patients were divided in two groups based on a normal (>1) or abnormal (<1) CPR (Table 2). The groups significantly differed in gestational age at the time of Doppler scan as well as at birth. Birth weight, birth weight percentile and head circumference were smaller in the abnormal CPR group. The use of antenatal steroids was significantly higher in the abnormal CPR group (82%) compared with cases with normal CPR (29%). All infants in the abnormal CPR group were delivered via caesarean section compared to 42% of infants with a normal CPR. Apgar scores and venous pH were also lower for infants with an abnormal CPR. Duration of admission to the neonatal ward/neonatal intensive care unit was significantly longer, and more often complicated by Respiratory Distress Syndrome or sepsis in the abnormal CPR group.

**Table 2:** Maternal and fetal/neonatal characteristics.

Patient Characteristics		Normal CPR			Abnormal CPR			P-value
**N**		24			17			
**Maternal**								
Age (years)		30.7	(23.2–40.6)		29.7	(23.6–39.5)		.930 ^a^
BMI (kg/m2)		22	(17.7–36)		24.4	(19.1–34.7)		.570 ^b^
Ethnicity (Caucasian)		19	(79.2)		16	(-94.1)		.373 ^d^
PE		3	(12.5)		4	(-23.5)		.421 ^d^
HELLP		3	(12.5)		0	(0)		.254 ^d^
PPROM		2	(8.3)		0	(0)		.502 ^d^
Oligohydramnios		15	(62.5)		10	(58.8)		.812 ^c^
Antenatal steroids		7	(29.2)		14	(82.4)		< .001 ^c^
(Stopped) smoking		12	(50)		7	(41.2)		.676 ^d^
Mode of delivery (SC)		10	(41.7)		17	(100)		< .001 ^c^
**Fetal/Neonatal**								
GA Doppler (weeks)		36.6	(27.1–39.2)		30.4	(25.6–37)		< .001 ^b^
GA Birth (weeks)		37	(28–40)		31	(26–37)		< .001 ^b^
Gender (Boys)		8	(33.3)		9	(52.9)		.335 ^c^
Birth weight (grams)		2075	(700–3035)		905	(560–1980)		< .001 ^a^
Birth weight z-score		-1.35	(-3.17–0.04)		-1.72	(-2.74 - -0.98)		.060 ^b^
Birth weight percentile		9	(1–52)		4	(1–16)		.045 ^b^
Head circumference (cm)		30	(23–34)		25.5	(21.2–32.5)		.004 ^b^
Apgar1’		8	(2–9)		6	(2–9)		.018 ^b^
Apgar5’		9	(5–10)		7	(4–10)		.008 ^b^
Apgar10’		9	(7–10)		8.5	(4–10)		.046 ^b^
Venous pH		7.28	(7.16–7.41)		7.25	(7.02–7.31)		.029 ^b^
Arterial pH		7.23	(6.88–7.35)		7.2	(7.04–7.26)		.195 ^b^
Base excess (mmol/l)		-6	(-10 - -1)		-4	(-11 - -2)		.867 ^a^
Duration of admission (days)		4.5	(0–48)		18	(2–90)		.007 ^b^
Complications								
	RDS	3	(12.5)		9	(52.9)		.007 ^c^
	BPD	1	(4.2)		3	(17.6)		.290 ^d^
	NEC	0	(0)		2	(11.8)		.166 ^d^
	IVH	0	(0)		3	(17.6)		.064 ^d^
	Sepsis	0	(0)		4	(23.5)		.024 ^d^
Mechanical ventilation		7	(29.2)		8	(47.1)		.328 ^c^

Characteristics given as median and (min-max) or frequency (%).

Abbreviations: Body Mass Index (BMI), Preeclampsia (PE), Hemolysis Elevated Liver enzymes and Low Platelets (HELLP), Preterm premature rupture of the membranes (PPROM), Cesarean Section (SC), Gestational age (GA), Respiratory Distress Syndrome (RDS), Bronchopulmonary Dysplasia (BPD), Necrotizing Enterocolitis (NEC), Intraventricular hemorrhage (IVH)

^a^ Student’s T-test

^b^ Mann-Whitney U Test

^c^ Chi-square Test

^d^ Fisher’s Exact Test.

### Average methylation of selected genes

We calculated the average methylation for each of the six genes (Table 3) after assessing the correlation coefficients (S2 Table) between individual CpGs within one gene. We observed a marginal non-significant trend toward hypermethylation on average (p = .095) for *VEGFA* in the abnormal CPR group. For *LEP* we found an average difference between the groups of 2.5% (95% CI: 0.5 to 5), (U = 118, z = -2.28, p = .023). Average methylation of *EPO*, *HIF1A*, *PHLDA2* and *DHCR24* did not differ between groups.

**Table 3:** Average methylation of the selected genes.

Gene	CpGs	Normal CPR		Abnormal CPR		P-value ^c^
*EPO*^*a*^	1–6	4.3	(3.4–6.0)	4.3	(3.3–7.7)	.979
*HIF1A*^*a*^	1–7	4.1	(2.5–5.3)	4.2	(2.9–5.6)	.347
*VEGFA*^*a*^	1–3	3.6	(3.0–5.4)	4.16	(3.1–5.1)	.095
*LEP*^*a*^	1–13	20.0	(13.2–31.6)	17.5	(10.1–26.4)	.023 *
*PHLDA2*^*a*^	1–4	16.3	(6.6–37.6)	17.5	(6.9–34.4)	.916
*DHCR24*^*b*^	1, 2, 4–8	7.2	(4.6–11.4)	7.2	(5.7–4.6)	.388

Values shown as median (range)

^a^ n = 24, n = 17

^b^ n = 22, n = 16

^c^ Mann-Whitney U Test

* significant at the .05 level (2-tailed)

### Methylation of individual *VEGFA and LEP* CpGs

**Fig 1:**
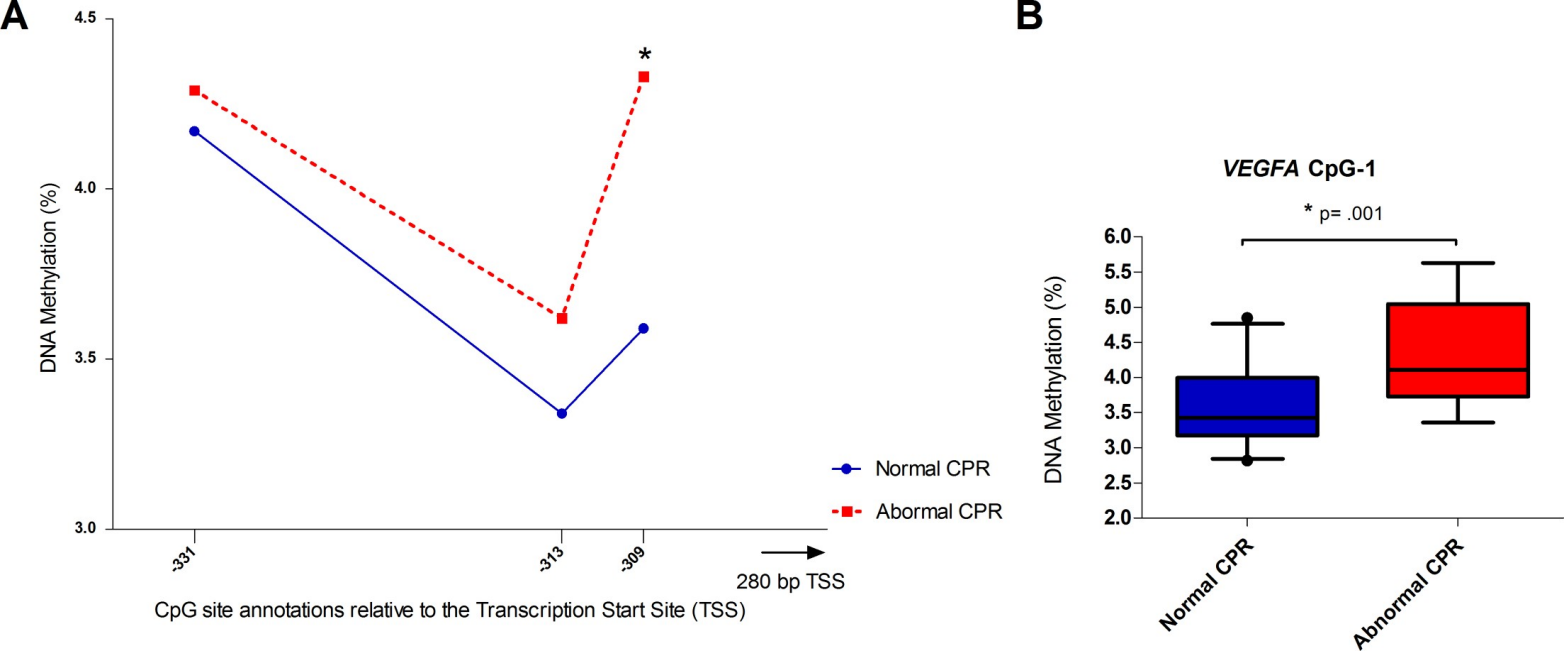
Methylation of *VEGFA* for normal and abnormal CPR. Mean shown for CpG-1 (-309) & CpG-2 (-313) and median for CpG-3 (-331). And C; boxplot with 5–95 percentile whiskers mean methylation of *VEGFA* CpG-1 (-309). Double asterisk (**) indicates significance of p <0.01.

**Fig 2:**
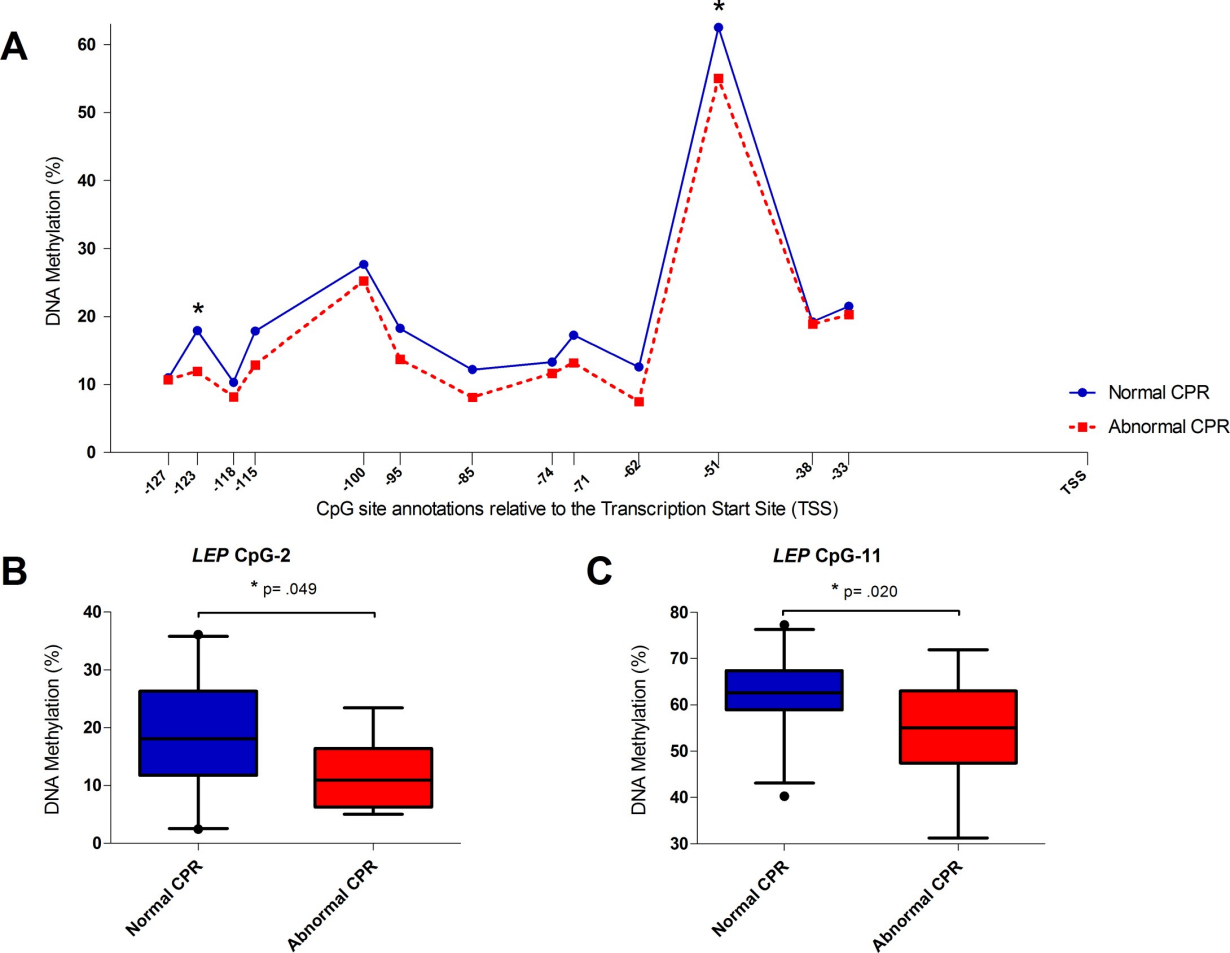
Methylation of *LEP* for normal and abnormal CPR. A; Methylation levels of *LEP* represented relative to the Transcription Start Site (TSS). CpG-1 (-127), CpG-2 (-123), CpG-3 (-118), CpG-4 (-115), CpG-5 (-100), CpG-6 (-95), CpG-7 (-85), CpG-8 (-74), CpG-9 (-71), CpG-10 (-62), CpG-11 (-51), CpG-12 (-38), CpG-13 (-33). Values are mean or median methylation as given in S1 Table. BC; boxplots with 5–95 percentile whiskers. B; Mean methylation of *LEP* CpG-2 (-123). Normal CPR (N = 20) Abnormal CPR (N = 14). C; Median methylation of *LEP* CpG-11 (-51). Asterisk (*) indicates significance of p <0.05.

For *VEGFA* and *LEP* we compared the median or mean differences, depending on the normality of the distribution, at the individual CpGs between the CPR groups. Because of the marginal trend toward significance for the average methylation of *VEGFA* we analyzed all 3 CpGs individually (Fig 1, S1 Table). We intended to also analyze another CpG locus on *VEGFA* in close proximity to the transcription site, but were unable to design primers of high enough quality for analysis. Of these sites, only CpG-1 (-309) methylation showed a statistically significant increase of 0.74% (95% CI: 0.328% to 1.15%) in the abnormal CPR group, t(39) = 3.635, p = .001 (Fig 1B). This is a medium effect size (Cohen’s d = 0.642). *LEP* showed a trend towards higher methylation in the normal CPR group at all CpGs (Fig 2, S1 Table). Of these, CpG-2 and CpG-11 were statistically significantly different between CPR groups. Mean methylation at CpG-2 was 5.96% (95% CI: 0.014 to 11.91) higher for normal CPR than abnormal CPR, t(32) = -2.042, p = .049 (Fig 2B) and median methylation at CpG-11 was 7.99% (95% CI: 1.18 to 14.52) higher (U = 116, z = -2.33 p = .020; Fig 2C).

### Association between CPR z-score and *VEGFA/LEP* methylation

**Fig 3:**
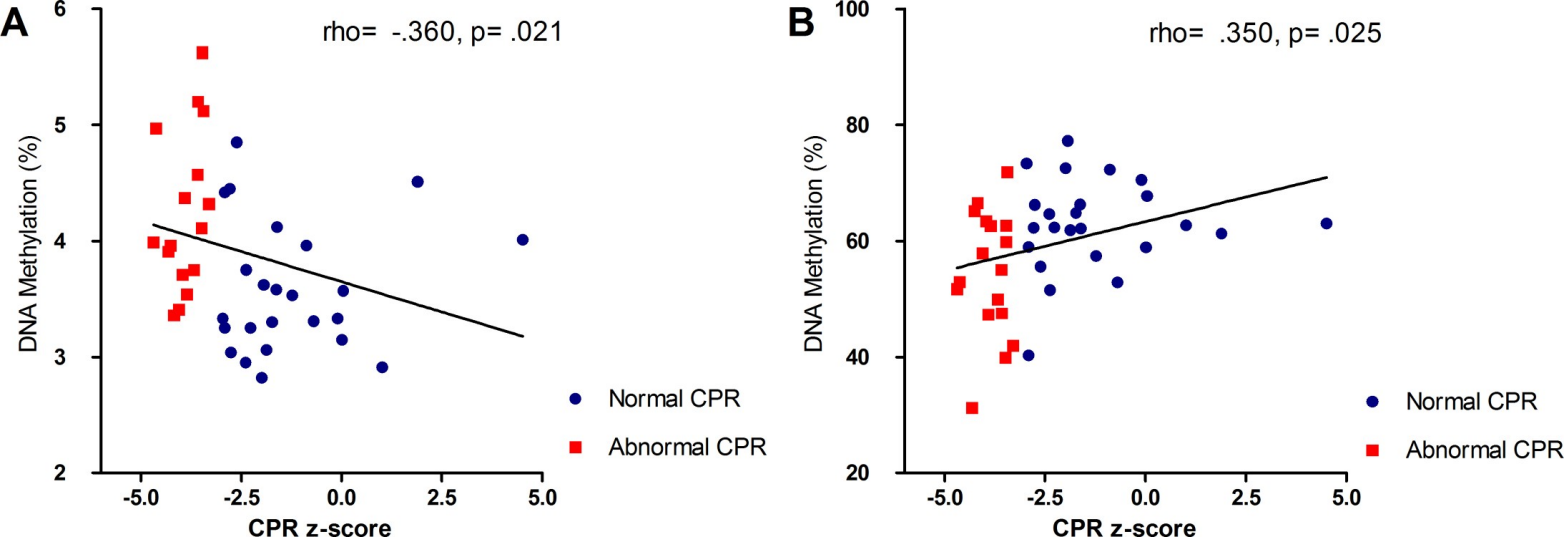
Spearman correlation coefficients (rho) and associated p-values provided for the association between and DNA methylation and CPR z-score. *VEGFA* CpG-1 (A), *LEP* CpG-11 (B). Asterisk (*) indicates significance of p <0.05.

To determine whether DNA methylation gradually changed as the CPR-z score deviated more from zero, we analyzed the association between the CPR z-score and methylation of *VEGFA* or *LEP* with Spearman's rank-order correlation. Because the CPR is not constant throughout gestation we used the CPR-z score. The average methylation of *VEGFA*, CpG-2, or CpG-3 did not correlate with the CPR z-score, however, *VEGFA* CpG-1 correlated negatively with CPR z-score, rho = -0.360, p = .021 (Fig 3A). The average methylation of *LEP*, and *LEP* CpG-2 did not correlate statistically significant (rho = .253, p = .11 and rho = .07, p = .693, respectively). *LEP* CpG-11 revealed a statistically significant positive correlation with the CPR z-score, rho (39) = .350, p = .025 (Fig 3B).

### Relationship between placental methylation CPR z-score, birth weight, and antenatal steroids

Because of the differences between the CPR groups in gestational age, birth weight z-score, and antenatal steroid usage we further investigated the influence of these parameters on the significantly altered CpGs: *LEP* CpG-2, *LEP* CpG-11 and *VEGFA* CpG-1.

Simple linear regression analyses did not reveal a relationship between methylation of *LEP* CpG-2 or *VEGFA* CpG-1 and gestational age, birth weight z-score, or antenatal steroid use. The CPR z-score predicted *LEP* CpG-11 methylation statistically significant, F(1, 39) = 4.699, p = .036, which explained 10.8% of the variance in methylation. Antenatal steroid use was associated with *LEP* CpG-11 methylation, F(1, 39) = 4.995, p = .031, and explained 11% of the variance in methylation. Gestational age was not associated with *LEP* CpG-11 methylation, F(1,39) = 2.498, p = .122. As gestational age was closely related to antenatal steroid use we decided not to enter gestational age in the multivariate model to avoid multicollinearity.

### Multiple linear regression analyses

Simple linear regression analysis showed a statistically significant relation between *LEP* CpG-11 methylation, CPR z-score and the use of antenatal steroids. Therefore, we entered CPR z-score and antenatal steroid use in a multiple regression model for the prediction of methylation. With both variables included, the predictive value of the model was statistically significant F(2,38) = 3.310, p = .047. This combined model explained 14.8% of the variance in *LEP* CpG-11 methylation.

## Discussion

Fetal programming might take place in response to the adverse in-utero environment such as in FGR, which has implications for the short-term as well as long-term health outcome. In this study, we investigated fetal programming of selected angiogenic and neurological genes. We analyzed methylation of *EPO*, *HIF1A*, *VEGFA*, *LEP*, *PHLDA2* and *DCHR24* in SGA placentas and found differential methylation of *VEGFA* and *LEP* depending on the presence of brain-sparing, defined as an abnormal CPR (<1). Average methylation levels of *EPO*, *HIF1A*, *PHLDA2* and *DCHR24* did not differ between SGA with or without an abnormal CPR. Comparison of average *VEGFA* methylation showed a marginal tendency towards significance between groups and CpG-1 (*VEGFA-*309) was significantly hypermethylated in the abnormal CPR group. For *LEP*, we observed an overall hypomethylation, and at the specific CpGs: CpG-2 (*LEP*-123) and CpG-11 (*LEP*-51) in the abnormal CPR group.

We found a decrease in methylation of 2.5% on average, 6% at CpG-2, and 8% at *LEP* CpG-11 in the abnormal CPR group. Methylation of CpG-11 is of special interest because this CpG is located at a binding site for CCAAT/enhancer binding protein-ɑ (CEBPA), a principal regulator of adipocyte differentiation and function [30]. Binding of CEBPA at this site highly activates transcription of *LEP* [31]. Methylation at this CpG results in transcriptional repression [29–31]. SGA infants with an abnormal CPR might thus have increased levels of Leptin in their placentas due to hypomethylation in the *LEP* region, specifically at CpG-11.

Recent studies have associated differential *LEP* expression and methylation with fetal growth, SGA, and neurodevelopmental outcome [6,7,15–19]. Lea et al. demonstrated less intense presence of Leptin in placentas from FGR singletons and growth-restricted infants in twin pregnancies [6]. Another study reported increased *LEP* expression in FGR [17]. Lazo-de-la-Vega-Monroy et al. support this by reporting elevated placental Leptin concentrations in SGA which was inversely correlated with placental weight [7]. In contrast to the elevated placenta levels, they showed that cord blood levels of Leptin and placental *LEP* receptor expression was lower in SGA, which both correlated positively with placental weight. Our findings are in line with the reported elevation of Leptin concentration [7,17], and is contradicting with decreased levels in SGA (9). Lesseur et al. investigated methylation of CpG-85 until CpG-33, as in the present study (CpG-7 to CPG-13), and did not observe an association between placental *LEP* methylation and birth weight [15]. They did find, however, an association between DNA methylation and *LEP* expression in their cohort. Disagreements between Lesseur et al. and our findings could result from our study-design. Our cohort consisted only of SGA cases and we compared methylation based on their CPR while Lesseur and colleagues compared placental methylation of SGA with appropriate-for-gestational age (AGA) fetuses.

Several potential confounders might have influenced our findings, as these were different between cases with abnormal and normal CPR, including antenatal steroids and gestational age at birth. Antenatal steroids were administered more often in the abnormal CPR group. Linear regression analyses revealed that both the CPR z-score and antenatal steroids were associated with *LEP* CpG-11 methylation. Both remained independent and significant predictors in the combined multivariate regression analysis. We were not able to discern exactly how much methylation was explained by which parameter in this small cohort because a large majority of the abnormal CPR group had received antenatal steroids. The observed hypomethylation and hypothesized elevated *LEP* expression could also be in line with the effects of antenatal betamethasone, as this is known to increase placental *LEP* concentrations [34]. Differences in methylation might also partly be explained by differences in gestational age between groups as it is known that gestational age is an important determinant of expression of leptin in placenta and of maternal leptin concentrations [35]. Placental *LEP* expression is higher and *LEP* receptors are less abundant in early pregnancy which could fit with the observed hypomethylation of *LEP* in the abnormal CPR group having a lower gestational age [36]. Therefore, we examined gestational age in our single and multiple regression analysis and found that this was not predictive of *LEP* CpG-11 methylation.

*VEGFA* was hypermethylated at CpG-1 in the abnormal CPR group. This specific CpG is a known binding site for *HIF1A*, an important pro-angiogenic factor which is beneficial in the acute response to hypoxia [13]. *VEGFA* is also a key-player in the anti-hypoxic response as it stimulates endothelial cell proliferation. This suggests that *VEGFA* is less expressed in the placenta of fetuses with an abnormal CPR because methylation can repress *VEGFA* expression [37]. The observed hypomethylation can be a sign of compensation to hypoxic stress in fetuses with a normal CPR, or that these fetuses were constitutionally small and not in a distressed and hypoxic state.

Differential methylation of *VEGFA* CpG-1 is of importance with regard to emerging preclinical research in placenta-directed gene therapy for FGR [38]. If this gene therapy would reach the clinical stage, assessment of *VEGFA* methylation in fetal cells derived from the maternal blood might be used to identify fetuses that could potentially benefit from placenta-directed gene therapy such as adenovirus vectors containing *VEGFA*. Again, the altered methylation status of *VEGFA* is the set-point of methylation throughout life which might predispose to the development of cardiovascular disease.

Our results suggest that adverse intrauterine conditions due to FGR and an abnormal CPR alter methylation of *LEP* and *VEGFA*. These altered methylation levels are a reflection of intrauterine conditions and represent the level of placental *LEP* and *VEGFA* during gestation. The placenta regulates its own growth and that of the fetus via *LEP*. Therefore, one might speculate that altered *LEP* levels are part of FGR pathophysiology. Altered *LEP* levels may dysregulate the development of important fetal endocrine feedback systems that regulate metabolism and adipose tissue differentiation. Furthermore, *LEP* may play a role in neuronal functional differentiation, glial development and development of the hippocampus and the cingulate cortex which might affect memory and cognition [39]. Altered *LEP* levels during fetal development might thus have an effect on memory, motor, and cognitive abilities.

Another function of placental *LEP* is immunomodulation which is essential for maintaining placental function at the interface between mother and her allogeneic fetus. With regard to immunomodulation, both hypomethylated *LEP* and hypermethylated *VEGFA* could contribute to low-grade inflammation. Although mostly known for its pro-angiogenic activity, *VEGFA* has an immunosuppressive role by inhibiting T-cell and dendritic cell development, and stimulating regulatory T-cells (Tregs) and myeloid-derived suppressor cells [40]. In contrast, *LEP* is a powerful pro-inflammatory hormone which can activate the innate and adaptive immune response [41]. *LEP* inhibits differentiation of Tregs and drives macrophage polarization to the inflammatory M1 phenotype. Extrapolating the hypo- and hypermethylation of *LEP* and *VEGFA*, respectively, this would suggest an increased set-point for *LEP* and decreased *VEGFA* levels in SGA fetuses with brain-sparing. These changes combined may predispose the infant and later adult, to a pro-inflammatory epigenetic profile that cannot easily be altered. Fetal programming may make ex-FGR infants susceptible to impaired neurodevelopment and a state of chronic low-grade inflammation, associated with metabolic syndrome, diabetes mellitus type 2 and cardiovascular disease [42].

Our study has several strengths. For the first time, we investigated methylation in an SGA cohort in relation to the CPR. This study is an extension of a well-designed prospective cohort study for which numerous clinical parameters were meticulously recorded [25]. Categorizing the SGA group depending on CPR allowed us to investigate methylation changes depending on severity of FGR and brain-sparing instead of the association between SGA and methylation in general. We adopted a gene specific approach and analyzed methylation levels of six gene promoter areas based on existing literature. Although this is a limited selection in comparison with genome-wide methylation profiling, this gave us the advantage of focusing on biologically relevant genes.

We also recognize some limitations. First of all, because the placentas were embedded in paraffin, we were not able to connect our methylation data with quantitative gene expression outcomes. Other researchers, however, have previously studied the association between DNA methylation and gene expression. The *VEGFA* region studied by us is a CG-rich promotor region in close proximity to the transcription start site which is associated with gene repression [43]. Induction of targeted DNA methylation of the *VEGFA* promoter by Siddique et al. demonstrates that *VEGFA* expression is repressed by increasing amounts of DNA methylation [37].

The association between *LEP* methylation and expression has also been thoroughly studied [31]. Negative associations with gene expression have been found in the maternal and fetal side of the placenta [18,44]. In line with this, methylation of a larger *LEP* promoter region in which our 13 investigated CpGs are located was also correlated with gene expression in the white adipose tissue visceral adipocyte fraction [45]. This relationship was explored further in a functional experiment where DNA methylation was inhibited in cultured human primary skin fibroblasts and HeLa cells which resulted in increased *LEP* expression [45].

The negative associations described here fit with the theory that increased gene promotor DNA methylation of *LEP* and *VEGFA* results in decreased expression. We expect that this relationship also exists within our cohort and assume that this could have an effect on gene expression.

Second, we studied a small sample size and the variation in gestational age at birth was great. Additionally, with this study design we cannot conclude whether the observed methylation changes are adaptive or maladaptive in the fetal period or thereafter, but the presence of a difference suggests that altered programming occurs in the placenta of FGR fetuses with an abnormally reduced CPR antenatally. Whether the observed differences in DNA methylation are sufficient to alter gene expression enough to cause a physiological effect could be explored further in an animal model of fetal brain-sparing.

## Conclusion

In conclusion, this study demonstrates that *LEP* and *VEGFA* are differentially methylated in SGA placentas, depending on the CPR. This suggests that the presence of FGR as defined by the CPR influences the epigenetic profile of the placenta differently. The increased risk of cardiovascular disease and metabolic syndrome are known consequences of FGR. Clearly, more research is needed to investigate the clinical significance of altered placental *LEP*, *VEGFA*, and other genes in relation to clinical care of growth-restricted children who will develop into adulthood. This study adds to the growing body of evidence regarding epigenetic programming in FGR. With increasing knowledge of the placenta and its epigenetic profile, the placenta can care for and protect the fetus throughout pregnancy and beyond birth by providing physicians with the methylation status at birth. This methylation profile might be used as a prognostic tool for targeted preventive interventions in the future.

## Supporting information

S1 Table Methylation of *VEGFA* and *LEP* per CPR group. TSS: location relative to the Transcription Start Site, Values shown as median (range) or mean (SE), ^a^ Normal CPR n = 24, Abnormal CPR n = 17, ^b^ Normal CPR n = 20, Abnormal CPR n = 14, * significant at the .05 level (2-tailed), ** significant at the .01 level (2-tailed), ^c^ Student’s T-test, ^d^ Mann-Whitney U Test.
(DOCX)

S2 Table Spearman’s rho correlation coefficients between individual CpGs within *EPO*, *HIF1A*, *VEGFA*, *LEP*, *PHLDA2* and *DHCR24*. * Correlation is significant at the .05 level (2-tailed). ** Correlation is significant at the .01 level (2-tailed).
(DOCX)
