# Supplementary material for: Differential placental DNA methylation of VEGFA and LEP in small-for-gestational age fetuses with an abnormal cerebroplacental ratio
Source: PLoS One. 2019 Aug 30;14(8):e0221972. doi: 10.1371/journal.pone.0221972 (PMC6716778; doi:10.1371/journal.pone.0221972)
Supplement: S1 Table — TSS: location relative to the Transcription Start Site, Values shown as median (range) or mean (SE), a Normal CPR n = 24, Abnormal CPR n = 17, b Normal CPR n = 20, Abnormal CPR n = 14, * significant at the .05 level (2-tailed), ** significant at the .01 level (2-tailed), c Student’s T-test, d Mann-Whitney U Test. (DOCX) [file pone.0221972.s001.docx]

**Table S1.** Methylation of *VEGFA* and *LEP* per CPR group.

| **Gene** | **CpG** | **TSS** | **Normal CPR** | | **Abnormal CPR** | | **p value** |
| --- | --- | --- | --- | --- | --- | --- | --- |
| ***VEGFA*** | CpG 1-3^a^ |  | 3.6 | (3.0-5.4) | 4.16 | (3.1-5.1) | .095 ^d^ |
|  | CpG-1^a^ | -309 | 3.59 | (0.11) | 4.33 | (0.18) | .001 ^c^** |
|  | CpG-2 ^a^ | -313 | 3.34 | (0.14) | 3.62 | (0.24) | .328 ^c^ |
|  | CpG-3 ^a^ | -331 | 4.2 | (2.4-8.9) | 4.3 | (2.9-6) | .895 ^d^ |
| ***LEP*** | CpG 1-13^a^ |  | 20.0 | (13.2-31.6) | 17.5 | (10.1-26.4) | .023* ^d^ |
|  | CpG-1^a^ | -127 | 11.0 | (2.3-39.8) | 10.7 | (2.3-19.3) | .874 ^d^ |
|  | CpG-2^b^ | -123 | 17.9 | (2.14) | 11.9 | (1.67) | .049* ^c^ |
|  | CpG-3^a^ | -118 | 10.3 | (3.5-37.6) | 8.2 | (4.6-15.3) | .138 ^d^ |
|  | CpG-4^a^ | -115 | 17.9 | (3.7-41.8) | 12.9 | (6.7-25.2) | .234 ^d^ |
|  | CpG-5^a^ | -100 | 27.7 | (9.5-56.9) | 25.2 | (16.3-40.2) | .146 ^d^ |
|  | CpG-6^a^ | -95 | 18.3 | (3-38.4) | 13.7 | (2.7-31.5) | .081 ^d^ |
|  | CpG-7^a^ | -85 | 12.2 | (2.5-30.9) | 8.1 | (2.9-18.9) | .552 ^d^ |
|  | CpG-8^a^ | -74 | 13.3 | (1.11) | 11.6 | (1.48) | .361 ^c^ |
|  | CpG-9^a^ | -71 | 17.3 | (4.1-26.5) | 13.2 | (4.1-28.4) | .475 ^d^ |
|  | CpG-10^a^ | -62 | 12.6 | (3.4-30.3) | 7.5 | (1.7-26.2) | .068 ^d^ |
|  | CpG-11^a^ | -51 | 62.5 | (40.3-77.3) | 55.0 | (31.2-71.9) | .020* ^d^ |
|  | CpG-12^a^ | -38 | 19.2 | (7.5-33.2) | 18.9 | (8.3-42.2) | .662 ^d^ |
|  | CpG-13^a^ | -33 | 21.5 | (5.4-31.9) | 20.3 | (5.5-43.5) | .937 ^d^ |

TSS: location relative to the Transcription Start Site

Values shown as median (range) or mean (SE)

^a^ Normal CPR n= 24, Abnormal CPR n=17, ^b^ Normal CPR n= 20, Abnormal CPR n=14

* significant at the .05 level (2-tailed), ** significant at the .01 level (2-tailed)


^c^ Student’s T-test, ^d^ Mann-Whitney U Test
