# Supplementary material for: Differential placental DNA methylation of VEGFA and LEP in small-for-gestational age fetuses with an abnormal cerebroplacental ratio
Source: PLoS One. 2019 Aug 30;14(8):e0221972. doi: 10.1371/journal.pone.0221972 (PMC6716778; doi:10.1371/journal.pone.0221972)
Supplement: S2 Table — * Correlation is significant at the .05 level (2-tailed). ** Correlation is significant at the .01 level (2-tailed). (DOCX) [file pone.0221972.s002.docx]

**Table S2.** Spearman’s rho correlation coefficients between individual CpGs within *EPO*, *HIF1A*, *VEGFA*, *LEP*, *PHLDA2* and *DHCR24.*

| **Gene** | **CpG** | | | | | | |
| --- | --- | --- | --- | --- | --- | --- | --- |
| ***EPO*** |  | **1** | **2** | **3** | **4** | **5** |  |
|  | **2** | .027 |  |  |  |  |  |
|  | **3** | -.115 | .012 |  |  |  |  |
|  | **4** | -.02 | -.296 | -.295 |  |  |  |
|  | **5** | .291 | -.023 | -.05 | .370* |  |  |
|  | **6** | -.034 | -.186 | .138 | .004 | -.123 |  |
| ***HIF1A*** |  | **1** | **2** | **3** | **4** | **5** | **6** |
|  | **2** | .125 |  |  |  |  |  |
|  | **3** | .128 | .345* |  |  |  |  |
|  | **4** | .253 | .456** | .369* |  |  |  |
|  | **5** | -.212 | .124 | .2 | .313* |  |  |
|  | **6** | .418** | .176 | .303 | .244 | .009 |  |
|  | **7** | .063 | .161 | .264 | .203 | .021 | .344* |
| ***VEGFA*** |  | **1** | **2** |  |  |  |  |
|  | **2** | .389** |  |  |  |  |  |
|  | **3** | 0 | .147 |  |  |  |  |
| ***LEP*** |  | **1** | **2** | **3** | **4** | **5** | **6** |
|  | **2** | .424* |  |  |  |  |  |
|  | **3** | .451** | .647** |  |  |  |  |
|  | **4** | .374* | .633** | .606** |  |  |  |
|  | **5** | .236 | .503** | .445** | .329* |  |  |
|  | **6** | .360* | .592** | .546** | .347* | .474** |  |
|  | **7** | .328* | .243 | .413** | .279 | .193 | .402** |
|  | **8** | .162 | .043 | -.007 | -.013 | -.001 | .222 |
|  | **9** | .172 | .002 | -.07 | -.12 | -.068 | .108 |
|  | **10** | .326* | .282 | .13 | .075 | .133 | .205 |
|  | **11** | .066 | .227 | .056 | .236 | -.079 | .102 |
|  | **12** | .242 | .094 | .098 | .12 | -.043 | .028 |
|  | **13** | .373* | .213 | .084 | .138 | -.047 | .008 |
|  |  | **7** | **8** | **9** | **10** | **11** | **12** |
|  | **8** | .127 |  |  |  |  |  |
|  | **9** | .176 | .559** |  |  |  |  |
|  | **10** | .327* | .586** | .369* |  |  |  |
|  | **11** | .09 | .118 | .151 | .186 |  |  |
|  | **12** | .071 | .430** | .438** | .455** | -.001 |  |
|  | **13** | .144 | .412** | .322* | .499** | .245 | .513** |
| ***PHLDA2*** |  | **1** | **2** | **3** |  |  |  |
|  | **2** | .122 |  |  |  |  |  |
|  | **3** | .077 | .854** |  |  |  |  |
|  | **4** | -.007 | .826** | .926** |  |  |  |
| ***DHCR24*** |  | **1** | **2** | **4** | **5** | **6** | **7** |
|  | **2** | .643** |  |  |  |  |  |
|  | **4** | .468** | .381* |  |  |  |  |
|  | **5** | .600** | .454** | .403** |  |  |  |
|  | **6** | -.086 | -.370* | -.016 | .026 |  |  |
|  | **7** | .661** | .451** | .518** | .355* | -.056 |  |
|  | **8** | .518** | .553** | .303 | .401* | .024 | .277 |

* Correlation is significant at the .05 level (2-tailed).

** Correlation is significant at the .01 level (2-tailed).
